# Clinical and epidemiological characteristics and outcomes of patients hospitalized for COVID-19 in Douala, Cameroon

**DOI:** 10.11604/pamj.2021.38.246.28169

**Published:** 2021-03-08

**Authors:** David Mekolo, Francois Adrien Bokalli, Fru McWright Chi, Steve Beukou Fonkou, Mbachan Maseoli Takere, Conrald Metuge Ekukole, Jean Moise Bikoy Balomoth, Dickson Shey Nsagha, Noel Emmanuel Essomba, Louis Richard Njock, Marcellin Ngowe Ngowe

**Affiliations:** 1 Intensive Care and Emergency Department, Laquintinie Hospital, Douala, Cameroon,; 2 Department of Medicine, Faculty of Health Sciences, University of Buea, Buea Cameroon,; 3 Department of Public Health and Hygiene, Faculty of Health Sciences, University of Buea, Buea Cameroon,; 4 Department of Surgery and Specialties, Faculty of Medicine and Pharmaceutical Sciences, University of Douala, Douala, Cameroon

**Keywords:** COVID-19, clinical characteristics, epidemiology, outcomes, Cameroon

## Abstract

**Introduction:**

the coronavirus disease (COVID-19) is a disease that originated from Wuhan in December 2019. It rapidly spread across the globe causing high mortality especially among the elderly. Africa though not spared has limited studies regarding its effects on its population. We therefore sought to describe the epidemiological and clinical characteristics of COVID-19 in Douala, Cameroon.

**Methods:**

we conducted a single-centre, retrospective, and observational study by reviewing records of patients managed for COVID-19 between the 8^th^ March 2020 and 31^st^, May 2020. Cases were confirmed by real-time reverse transcriptase - polymerase chain reaction and were analysed for epidemiological, demographic, clinical, and radiological features. Outcomes were either clinical improvement by Day-28 or in-hospital mortality.

**Results:**

we analyzed 282 case files, 192 were males (M: F=2: 1). The mean age was 52 (+/- 15) years. Hypertension and diabetes accounted for 75% of the chronic medical conditions identified. Main presenting complaints were dyspnea, cough, asthenia, and fever (55-60%). Radiographic analysis showed a ground-glass appearance in 85% of cases. Chloroquine/Hydroxychloroquine was the most (91.8%) frequently used drug in management protocols, 35% needed oxygen supplementation while 6 patients were intubated. Severe pneumonia (11.3%) was the commonest complication. They were 91 admissions in the intensive care unit. The average length of hospital stay was 10 (+/- 5) days. The mortality rate was 32%.

**Conclusion:**

our findings are concordant with universally reported data of COVID-19 hospitalised patients. These parameters are essential in designing effective prevention and control programs aimed at reducing the impact of the COVID-19 pandemic particularly in countries with limited resources.

## Introduction

A coronavirus disease also called coronavirus respiratory syndrome, coronavirus pneumonia, coronavirus flu, or any other variant is a disease caused by members of the coronavirus (CoV) family. These include: Severe Acute Respiratory Syndrome (SARS), Middle East Respiratory Syndrome (MERS), and coronavirus disease 2019 (COVID-19) [1]. MERS-CoV has caused more than 10000 cumulative cases in the past two decades with mortality rates of 37%, while for SARS-CoV it was 10% [2]. In December 2019, Wuhan city, the capital of Hubei province in China, became the centre of an outbreak of pneumonia of unknown cause. By January 7^th^, 2020, Chinese scientists had isolated a novel coronavirus, severe acute respiratory syndrome coronavirus 2 (SARS-CoV-2; previously known as 2019-nCoV), from these patients with virus-infected pneumonia which was later designated coronavirus disease 2019 (COVID-19) in February 2020, by World Health Organization (WHO) [3].

The 2019-20 coronavirus outbreak was declared a pandemic by the WHO on 11^th^ March 2020. As of April 1^st^, 2020, a total of 823626 cases had been documented globally and 40598 deaths. Europe was the most affected region with 56.4% of cases and 74.1% of deaths, while, Africa had recorded 4073 cases for 91 deaths [4]. In Cameroon, the first case was reported on March 6^th^, 2020, and within days declared a state of emergency that included travel bans, lockdowns, widespread testing, and quarantine [5]. As of May 30^th^, 2020, the Ministry of Public Health who regularly communicated on figures at a national level, said 5659 persons tested positive, 185 deceased and 3441 recovered cases [6].

The clinical spectrum of SARS-CoV-2 infection appears to be wide, encompassing asymptomatic infection, mild upper respiratory tract illness, and severe viral pneumonia with respiratory failure and even death [7]. Pneumonia mostly occurs in the second or third week of symptomatic infection. Prominent signs of viral pneumonia include decreased oxygen saturation, blood gas deviations, changes visible through chest X-rays and other imaging techniques, with ground glass abnormalities, patchy consolidation, alveolar exudates, and interlobular involvement, eventually indicating deterioration. Blood analysis showed lymphopenia to be common, and inflammatory markers are elevated [8, 9]. Studies from countries that were first affected reported higher mortality in specific patient groups including: elderly patients, patients with cardiovascular and respiratory comorbidities [10, 11]. Given the rapid spread of COVID-19, we aimed to describe epidemiological, clinical, radiological characteristics and outcomes of confirmed COVID-19 patients, admitted in the Laquintinie hospital Douala, Cameroon.

## Methods

**Study design and period:** this study was a hospital-based descriptive cross-sectional retrospective study carried out for three months (March to May 2020).

**Study area:** the study was carried out in Douala, the economic capital of Cameroon, which has the busiest international airport in the country. It is a cosmopolitan city, recording the largest number of confirmed cases in the country as of the date this research was conducted. It is a town of about 3.7 million inhabitants as of 2015 [12]. The Laquintinie Hospital of Douala (LHD) was the designated focal point for reception, treatment, and isolation of COVID-19 cases in the Littoral region. It is a tertiary hospital dispensing extensive medical services and a very important health structure in the country due to the affordability of its wide range of services, seniority, and surface area. It also serves as a teaching hospital. Along with the well-equipped 24-hour casualty unit, there exist laboratory, pathology, and radiologic units. The Intensive Care Unit (ICU) has a 12-bed capacity, with two ventilators and the unit is managed by three anaesthesiologists. Two 100 bed capacity wards were set aside for isolation of asymptomatic patients and those with mild to moderate presentations of COVID-19, managed by 40 health personnel among which infectious disease specialists, cardiologists, pneumologists, and general practitioners.

**Study population and sample:** subjects for the study included all files of COVID-19 patients who were admitted in LHD from 1^st^, March 2020 to 31^st^, May 2020.

**Inclusion criteria:** all confirmed cases of COVID-19 admitted and managed in LHD during the study period with complete data in files.

**Exclusion criteria:** cases not meeting the inclusion criteria.

**Sample size:** all those who met the inclusion criteria were included.

**Study procedures:** authorization to carry out this research was obtained from the director of the Laquintinie Hospital. Ethical clearance was equally sought and gotten from the Institutional Ethics Committee (IEC) of the hospital. This permitted us to use both the electronic and physical files of patients consulted and managed in the hospital during the study period. Anonymity was respected by coding the files and guaranteed that patient´s names or identification were to be disclosed on any study document. Then proceeded to the various wards to have access to the records of patients treated for this condition. We obtained epidemiological, demographic, clinical, laboratory, management, and outcome data from patients´ medical records. Clinical outcomes were followed up to 28 days following admissions.

Laboratory confirmation of COVID-19 was being done initially by the Centre Pasteur du Cameroun, a reference biological laboratory in the country, then by the hospital laboratory. Nasopharyngeal and oropharyngeal swab samples were collected following standard safety procedures. The analysis was done by the real-time reverse transcriptase-polymerase chain reaction (RT-PCR) for suspected cases following the protocol established by the country which was adapted from WHO guidelines [13]. In certain emergency cases with high radiological suspicion alongside clinical evaluation were considered and treated as COVID positive cases similar to situations in other western countries [14]. Radiological interpretations of the chest computed tomography (CT) scans were done by radiologists. The cases were classified in clinical syndromes as described by WHO in their interim guidance of March 2020 [13]. In cases of incomplete data from the records, we obtained data by direct communication with attending physicians and other healthcare providers. All data were checked by two physicians.

**Data management and analysis:** data were entered and analysed using SPSS version 25.0. All data collected were coded and stored in a computer hard drive. Numerical variables were presented as means (Standard Deviation (SD)) and/or median (Interquartile range (IQR)) where appropriate, or as frequencies (n) and percentages (%) after categorizing using predefined cut-offs or the median. Categorical variables were presented as frequencies (n) with proportion (% and 95% Confidence Interval (95%CI)). Comparisons for numerical variables were mean or median differences. Results were represented in tables and figures, to ease visualization and comprehension. An alpha value was set at P=0.05 and the confidence interval at 95% CI.

## Results

**Figure 1:**
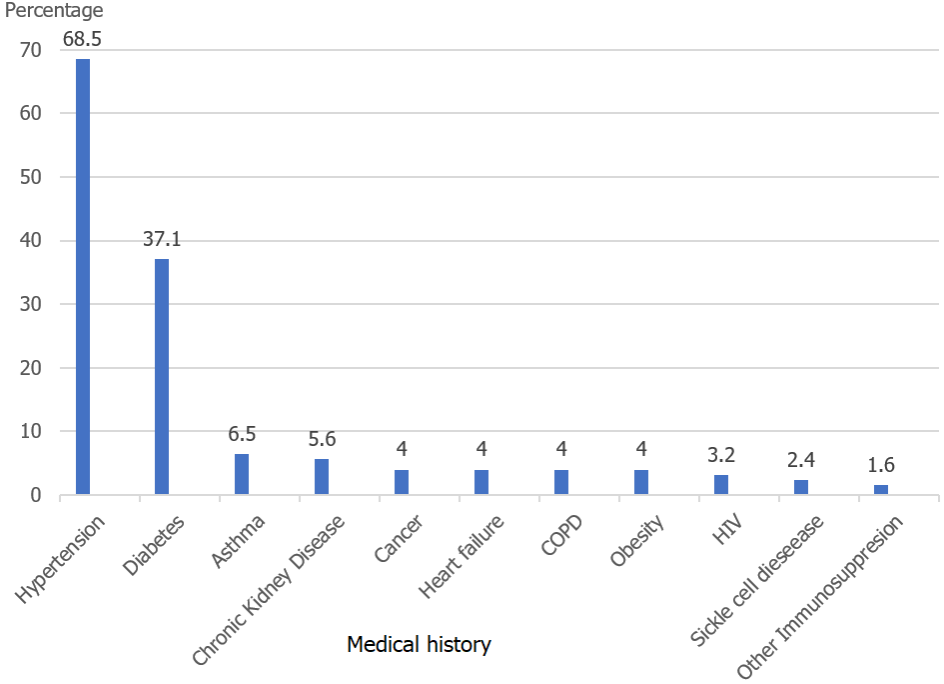
medical history of the study population

**Figure 2:**
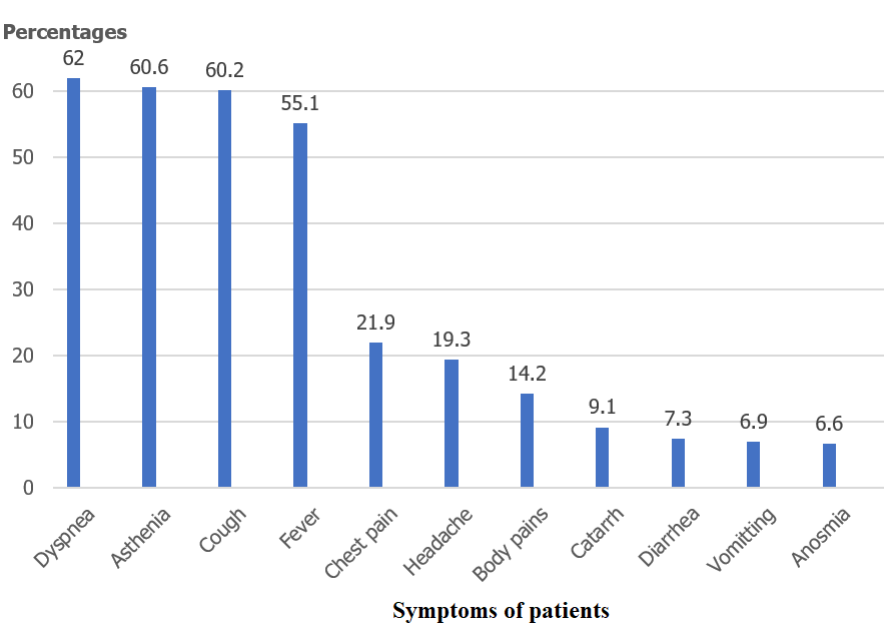
presenting symptoms of patients on admission

Of the 379 patients admitted to the hospital within the study period, files regarding 282 of them were retrieved and analysed for our study. The others being either lost to follow up had incomplete/empty files or no clear reference to confirmatory COVID-19 diagnosis. Of the 282 cases, 192 (68.1%) were males giving an M: F ratio of 2: 1. The mean age was 52 (+/- 15) years. The age range 40-59 years had the highest population (Table 1). We identified at least one chronic medical condition in 124 cases (44%), with hypertension and diabetes accounting for about 75% of these conditions identified (Figure 1). Two hundred and seventy-four (97.2%) cases reported at least one of the major symptoms investigated with fever, cough, dyspnea, and asthenia, and chest pain, contributing to 80% of these symptoms. Dyspnea (62%), fever (60%), and cough (60%) were mostly experienced concomitantly by our cases (Figure 2). The median duration of symptoms before presentation at the hospital was 5 days (IQR 3 - 10).

**Table 1:** characteristics of study population

Variables		Frequency	Percentage
**Age (years)**			
<20		2	0.7
20-39		61	21.6
40-59		125	44.3
60+		94	33.3
**Gender**			
Male		192	68.1
Female		90	31.9
**Clinical syndromes**			
Mild illness		139	49.2
Moderate illness		53	18.8
Severe pneumonia		32	11.3
Sepsis		26	9.2
ARDS		24	8.5
Septic shock		8	2.8
**Medical Therapy**			
Chloroquine/Hydroxychloroquine		259	91.8
Antibiotics		240	85.1
Corticosteroids		62	22.0
Lopinavir/Ritonavir		6	2.1
Oxygen therapy		100	35.4
Mechanical ventilation	Non invasive	81	28.7
	Invasive	6	2.1

ARDS: Acute Respiratory Distress Syndrome

Most patients (49%) developed a mild or uncomplicated form of the disease, while a few had severe presentations that required immediate intensive care. The commonest severe manifestation was severe pneumonia (11.3%) closely followed by sepsis and acute respiratory distress syndrome respectively (8.5% and 9.2%). Table 1 shows the various clinical syndromes shown by the patients as described by the WHO. Patients benefited from a first CT scan evaluation, averagely the first day of hospitalization (Mean (SD): 0.5 (0.0) days from admission). According to chest CT scan findings 240/282 (85%), patients showed ground-glass opacities while just 29 (10.2%) had patterns typical of alveolar condensation. Additionally, pulmonary embolism occurred in one patient. The percentage of lung involvement in CT scan findings was 10-25% in 35.9% of cases making it the most frequent observation while a lung involvement of >75% occurred in just 5.2% of cases. As the pandemic evolved, treatment protocols were continuously revised, and no single treatment protocol was continued throughout the entire 3 months for this series of patients. Anticoagulation with low-molecular-weight heparin (LMWH), vitamin C and Zinc were given as a standard of care to all patients. The dose of LMWH was dependent on disease severity. Other symptomatic treatments were given as judged necessary by the medical team. All patients were treated in isolation.

The proportion of cases put on oxygen was 35.5%, often within the first day of hospitalization (Mean (SD): 0.8 (0.0) days). The average duration of stay on oxygen was 2.5 (+/- 2) days or 60.5 (+/- 48) hours. Ninety-four patients were put on non-invasive mechanical ventilation, with the mean duration per patient of 3.2 days (SD +/- 2.0). Six patients were intubated to assist ventilation due to increasing severity. However, only one of the patient´s survived following intubation as the late presentation, as well as the severity of cases, proved fatal to the others. A summary of different therapies prescribed is shown in Table 1. By the end of the study period, the mean duration of hospital stay was 10 days (with an SD of 5.2). 91 patients were admitted to the ICU due to the severity of their illness or non-improvement despite oxygen supplementation. The patients were discharged following therapy and a negative test was obtained. We had a mortality of 32% (91/282), 42% of whom died in the intensive care unit. For cases that died, the average duration from the date of admission to death was 4.4 (+/- 3.0) days (Table 2).

**Table 2:** outcome of patients with COVID-19

Variables		
Outcome	Frequency	Percentage
Admitted in ICU	91	32
Discharged	191	68
Death	91	32
**Length of hospital stay/days**	**Mean**	**Standard deviation**
Discharged patients	10	5.2
Deceased patients	4.4	3

## Discussion

This study is among the first to report epidemiological, clinical characteristics, and outcomes of hospitalized COVID-19 patients in Cameroon. The few number of cases recorded could be explained by the fact that many patients preferred home isolation and other private health facilities and were not included in this study. None of our patients had a history of contact with Wuhan seafood or recent travel to China, this points more to a human-to-human transmission of the virus. There was a male predominance in our study, this was similar to other studies [11, 12]. The mean age was 57 years, similar to studies in diverse geographical areas [15-18] suggesting that middle-aged and elderly people were more susceptible to infection, whereas healthy, young adult patients were less susceptible. Gebremariam *et al*. [19] in Ethiopia however had a younger population. This could be explained by the fact that more than 50% of the cases had a travel history out of the country supposing they are a more active and therefore young population.

Among the comorbidities evaluated, hypertension and diabetes were seen in the more severe presentations and usually had a poorer prognosis for COVID-19, they were also the commonest comorbidities detected in patients. These were also the highest comorbidities in studies by Zamparini *et al*. and Liu *et al*. [20, 21]. The prevalence of HIV in our cohort was 3.2%, which is similar to that seen in the general population in Cameroon (3.7%) [22]. However, a larger cohort of HIV-positive patients is needed to assess the association between HIV and COVID-19. Cummings *et al*. and Nachega *et al*. had similar low observations [15, 17]. Dyspnoea, cough, and fever were the most prominent symptoms as compared to those of the digestive and nervous systems. As the pandemic evolved, more symptoms were reported concerning the disease and as such, the frequency of the symptoms was observed based on what new findings the science world had found. The major presentation of patients was similar to studies done elsewhere [21, 23, 24].

Although the median duration of symptoms was 5 days similar to other studies [6, 25], the symptoms being unspecific could explain why patients took a certain number of days before presenting at a hospital. We noticed the wide disparity in the range (1 to 51 days), which could be explained by the fact that two populations existed. Those with increased fear of the disease who presented immediately they felt any symptoms as described by the extensive media coverage and those who denied its existence and therefore stayed away from hospitals and only presented very late after the onset of symptoms when it was quite severe, as further elicited by some studies [26-29]. Ground glass opacity was the most frequent CT scan pattern seen in our patients as observed by Long *et al*. in his study [30]. The ground-glass opacity pattern always agreed with biological results. The percentage of lung involvement was usually small as most patients did the CT scan on entry or did so early and were most of the time mild cases. The larger the lung involvement the greater the probability of poor prognosis.

The criteria for discharge were good clinical evolution and negative PCR COVID-19 test. In our study, on average 10 days following admission were necessary for change of test results from positive to negative coupled with good clinical evolution. In South Africa [21] however, it was 6 days and in China it was 12 days [6]. This could be explained by the difference in retesting protocols in these various hospitals. Up to 42% of the patients within the study period were admitted to the ICU, this is quite high when compared to other studies [13, 19, 31]. This could be because during the early phases our study site was the sole hospital in the region receiving COVID-19 patients therefore referrals were mostly of serious cases requiring intensive care. Our mortality rate was 32%, fewer than half of which were in the ICU. It was higher than the mortalities of other studies [7, 21] and this could be explained by the absence of ventilators and a few beds in the ICU. Also, the fact that there are currently no effective drugs that can target COVID-19. Therefore, the treatment was focused on symptomatic and respiratory support.

**Study limitations:** the retrospective nature of this work made it difficult to retrieve all the necessary information for this research. This study did not include patients who might have probably be found in other facilities and thus might have a lower prevalence. Delay or absence of COVID-19 diagnostic kits made prompt diagnosis a major challenge thereby leaving a lot of suspected but not confirmed cases in doubt and therefore not used in our analyses. With the resources available, an extensive workup could not be done, however effective management of these resources helped in saving many lives. The confirmed cases of COVID-19 might not reflect the actual number of persons infected by COVID-19 as many cases were asymptomatic and some went untested further to the limited testing resources in the country.

## Conclusion

COVID-19 has spread rapidly since it was first identified in Wuhan and has been shown to have a wide spectrum of severity. This study is among the first to provide information on the epidemiological and clinical characterization of the COVID-19 pandemic in Cameroon. In our study, the male population was more affected and those of the 5^th^ and 6^th^ decade of life presented most at the hospital. The patients presented a variety of symptoms and were mostly mild illnesses. Ground glass appearance was frequently observed in chest imaging. The mortality rate among hospitalized patients was high. Our study provides insights into COVID-19 manifestations in Cameroon, more data are needed with larger cohort observational studies to better define the epidemiology and factors affecting COVID-19 outcomes. Continuous monitoring and tracking are required to secure an in-depth understanding of the disease, thereby providing an improved evidentiary basis for standardizing the diagnosis and treatment of COVID-19.

### What is known about this topic

- It is a global pandemic with elevated mortality worldwide;
- Sub-Saharan Africa was quite unaffected compared to other parts of the world.

### What this study adds

- It gives an insight of the early days of the pandemic in the country´s main hospital;
- It describes the epidemiological and clinical characteristics of patients admitted.
